# Trans-ancestry polygenic models for the prediction of LDL blood levels: an analysis of the United Kingdom Biobank and Taiwan Biobank

**DOI:** 10.3389/fgene.2023.1286561

**Published:** 2023-11-23

**Authors:** Emadeldin Hassanin, Ko-Han Lee, Tzung-Chien Hsieh, Rana Aldisi, Yi-Lun Lee, Dheeraj Bobbili, Peter Krawitz, Patrick May, Chien-Yu Chen, Carlo Maj

**Affiliations:** ^1^ Luxembourg Centre for Systems Biomedicine, University of Luxembourg, Esch-Sur-Alzette, Luxembourg; ^2^ Institute for Genomic Statistics and Bioinformatics, University of Bonn, Bonn, Germany; ^3^ Taiwan AI Labs and Foundation, Taipei, Taiwan; ^4^ Center for Computational and Systems Biology, National Taiwan University, Taipei, Taiwan; ^5^ Department of Biomechatronics Engineering, National Taiwan University, Taipei, Taiwan; ^6^ Center for Advanced Computing and Imaging in Biomedicine, Natinal Taiwan University, Taipei, Taiwan; ^7^ Centre for Human Genetics, University of Marburg, Marburg, Germany

**Keywords:** polygenic risk score, East Asia, Taiwan Biobank, United Kingdom Biobank, LDL cholesterol

## Abstract

Polygenic risk score (PRS) predictions often show bias toward the population of available genome-wide association studies (GWASs), which is typically of European ancestry. This study aimed to assess the performance differences of ancestry-specific PRS and test the implementation of multi-ancestry PRS to enhance the generalizability of low-density lipoprotein (LDL) cholesterol predictions in the East Asian (EAS) population. In this study, we computed ancestry-specific and multi-ancestry PRSs for LDL using data obtained from the Global Lipid Genetics Consortium, while accounting for population-specific linkage disequilibrium patterns using the PRS-CSx method in the United Kingdom Biobank dataset (UKB, n = 423,596) and Taiwan Biobank dataset (TWB, n = 68,978). Population-specific PRSs were able to predict LDL levels better within the target population, whereas multi-ancestry PRSs were more generalizable. In the TWB dataset, covariate-adjusted *R* ^2^ values were 9.3% for ancestry-specific PRS, 6.7% for multi-ancestry PRS, and 4.5% for European-specific PRS. Similar trends (8.6%, 7.8%, and 6.2%) were observed in the smaller EAS population of the UKB (n = 1,480). Consistent with *R* ^2^ values, PRS stratification in EAS regions (TWB) effectively captured a heterogenous variability in LDL blood cholesterol levels across PRS strata. The mean difference in LDL levels between the lowest and highest EAS-specific PRS (EAS_PRS) deciles was 0.82, compared to 0.59 for European-specific PRS (EUR_PRS) and 0.76 for multi-ancestry PRS. Notably, the mean LDL values in the top decile of multi-ancestry PRS were comparable to those of EAS_PRS (3.543 vs. 3.541, *p* = 0.86). Our analysis of the PRS prediction model for LDL cholesterol further supports the issue of PRS generalizability across populations. Our targeted analysis of the EAS population revealed that integrating non-European genotyping data with a powerful European-based GWAS can enhance the generalizability of LDL PRS.

## Background

Blood lipid levels are significant, modifiable, and heritable risk factors for coronary artery disease (CAD), including low-density lipoprotein cholesterol (LDL-C) (Nelson, 2013). Previous studies have shown that lipid levels have moderate-to-high heritability variations of up to 60% (Weiss et al., 2006; Kathiresan et al., 2007). Numerous common variants have been discovered in recent genome-wide association studies (GWASs) associated with LDL and many other traits (Sollis et al., 2022). However, the majority of these variants are weakly associated individually with a given trait or disease and have limited predictive power. The cumulative effects of several common variants have been suggested to contribute significantly to the risk stratification for clinical utility. Methods have been developed for analyzing data from these large-scale studies and detecting genetic variants and phenotype associations, and one such method is the polygenic risk score (PRS). Several studies have evaluated the association between PRS and the risk of various conditions (Khera et al., 2018), including lipid traits (Graham et al., 2021), CAD (Fahed et al., 2020), cancer (Hassanin et al., 2022; Hassanin et al., 2023), diabetes (Dornbos et al., 2022), and neurodevelopmental disorders (Nalls et al., 2019).

One of the major issues concerning the translational use of PRS is the strong dependency on population specificity. In fact, the performance of PRS can significantly be influenced by the linkage disequilibrium (LD) across variants and allele frequencies that are specific to different populations (Ding et al., 2023). As a consequence, PRS has mostly been limited to European ancestry cohorts for which larger reference GWASs are available (Duncan et al., 2019). In addition to LD and allele frequencies, gene–environment (Ordovas and Shen, 2008) interactions may also be responsible for different genetic susceptibilities toward a trait. Since individuals with East Asian ancestry account for more than a fifth of the global population, understanding genetic variations in the East Asian population is crucial to improve risk characterization and preventive interventions (Ge et al., 2022).

In the last few years, the availability of large population-based cohorts and cross-ancestry GWAS also enabled the development of novel computational algorithms to improve the generalizability of PRS (Ruan et al., 2022; Hoggart et al., 2023). A multi-ancestry, GWAS meta-analysis of lipid levels was conducted by the Global Lipid Genetics Consortium, including 350,000 people of non-European ancestry, 150,000 East Asian individuals, and approximately 1.65 million people worldwide (Graham et al., 2021). The study also helped improve our understanding of the genetic component associated with lipid levels by increasing diversity rather than including additional European ancestry individuals.

In this study, we derived ancestry-specific and cross-ancestry PRS to predict the serum LDL level by first considering all populations and then focusing on East Asian individuals. Particularly, we derived six LDL-PRSs: four ancestry-specific PRSs (East Asian, South Asian, European, and African) and two multi-ancestry PRSs (East Asian with European meta-analysis and the four ancestry meta-analyses). The six PRSs were tested among nine population groups estimated from the United Kingdom Biobank (UKB, n = 423,596). We focused on the East Asian ancestry group from the UKB and validated PRS with participants from the Taiwan Biobank (TWB, n = 68,978). Then, we tested the associations between PRS and LDL cholesterol changes among East Asian individuals in both biobanks.

## Methods

### Study subjects

The analysis was performed using genetic and phenotypic data of the UKB and TWB. The UKB is a population-based cohort study, with over 500,000 individuals aged 40–69 years at the time of recruitment. We used the available imputed genotype array data through the UKB (Bycroft et al., 2018). We excluded outliers with high genotype missing rates, putative sex chromosome aneuploidy, and discordant reported sex vs. genotypic sex (Hassanin et al., 2021). We randomly excluded one from each pair of related individuals if the genetic relationship was closer than the second degree, defined as kinship coefficient >0.0884 as calculated by the UKB. A previous approach was applied to divide UKB individuals into nine ancestry groups by projecting data onto the principal component analysis (PCA) space of 1,000 Genomes Project (Privé et al., 2022).

The TWB is a Taiwanese-based cohort study, with 68,978 individuals aged 30–75 years across 750 k SNPs (Wei et al., 2021). For more overlapping SNPs with PRS models, we imputed the TWB cohort. First, we filtered out SNPs based on certain criteria: a missing rate of 0.2 for variants, missing rate of 0.5 for samples, and Hardy–Weinberg equilibrium of 5 × 10^−7^. Subsequently, we employed SHAPEIT4 and IMPUTE5 to impute the genotype with a reference based on the whole-genome sequencing data of 1,496 Taiwanese individuals. SNPs with a maximum genotype probability of less than 0.2 were removed. In total, we obtained 15 million SNPs for 69 k Taiwanese individuals as our external validation set.

### United Kingdom Biobank ancestry grouping

We assigned the samples to different countries using PC-projection, as demonstrated in a previous study (Privé et al., 2022). In this previous study, the authors explored different methods to classify individuals into ancestry groups using the PCA of genome-wide genotype data. They found that Euclidean distances in the PCA space are proportional to the genetic differences between populations and recommend using this distance measure. They suggest using all principal components to capture the population structure, as using only two or four is insufficient for distinguishing certain populations. They applied PCA-based distance to infer ancestry in datasets and proposed two solutions: projecting PCs to reference populations or using internal data. They demonstrated that these solutions are effective for inferring ancestry and grouping genetically similar individuals. Here, we used this approach to define the nine ancestry groups based on United Kingdom Biobank data and birth country information. These groups encompassed a range of geographical and ancestral backgrounds, with some individuals from neighboring countries. In particular, the defined ancestry groups were as follows: East Asian, using China as the center; European, using three different centers of United Kingdom, Italy, and Poland; African, using dual centers in Nigeria and the Caribbean; South Asian, using India as the center; Middle East, centered on Iran; and Ashkenazi Jewish, representing individuals with Ashkenazi Jewish ancestry.

Furthermore, given that the majority of TWB individuals clustered with the Han Chinese South group (Chen et al., 2016), we employed a complementary approach, to further explore East Asian subpopulations within the UKB dataset. We projected UKB samples into principal component space based on the five East Asian subpopulations from the 1,000 Genomes Project as reference points. We only used two East Asian subpopulations from the UKB (Han Chinese South [CHS] and Kinh in Ho Chi Minh City, Vietnam [KHV]) and excluded the other three East Asian subpopulations due to sample size limitations.

### Construction of multi-ancestry polygenic score

To evaluate the potential of PRS to predict increased LDL cholesterol levels in East Asian ancestry, we used the latest GWAS that was conducted in different populations to derive an ancestry-specific or multi-ancestry LDL PRS (Graham et al., 2021). We considered the summary statistics that did not include United Kingdom Biobank samples. Six PRSs were created: one for each ancestry (East Asian, South Asian, European, and African) and two meta-analyses using multi-ancestry GWAS (one using East Asian and European ancestry and the other using the four ancestries). PRS weights were conducted using PRS-CSx (Ruan et al., 2022) (accounting for population-specific allele frequencies and LD patterns) and the 1000 Genomes Project as a reference panel that matched the ancestry of each discovery GWAS. The PRS-CSx method incorporates summary statistics from different GWASs and links the genetic effects across populations using a continuous shrinkage prior to that being shared between them. This approach allows for a more precise estimation of effect sizes by using information from the summary statistics and taking advantage of the variation in linkage disequilibrium across the discovery samples. By jointly modeling these multi-ancestry summary statistics, PRS-CSx may be able to better capture the underlying genetic effects and produce more accurate predictions. We developed the multi-ancestry PRS using the “--meta” option provided by the software. We tested each of the six PRSs in the nine population groups from the UKB. Then, we evaluated the six PRSs among the East Asian cohort of the TWB. We compared the PRS performance between individuals in the TWB and two East Asian subpopulations from the UKB (CHS and KHV) from the 1000 Genomes Project.

### Assessment of PRS accuracy

We assessed the prediction accuracy of the six PRSs in the nine estimated populations from the UKB and Taiwanese population from the TWB. We standardized PRSs to a mean of 0 and standard deviation of 1. In the evaluation of PRS and their impact on the prediction of LDL levels, we considered the increase in explained variance (incremental *R* ^2^) due to PRS. The following outlines the procedure: two models were utilized in our analysis.

(1) Full model: This model incorporated PRS as an additional predictor, along with other covariates, including sex, age, age^2, and the first four genetic principal components (formula: LDL ∼ PRS + sex + age + age^2^ + PC1 + PC2 + PC3 + PC4).
(2) Reference model: In contrast, the reference model considered only the covariates without PRS (formula: LDL ∼ sex + age + age^2^ + PC1 + PC2 + PC3 + PC4). To calculate the incremental R2, we performed linear regression for both models. Incremental R2, as performed in previous studies (Huang et al., 2022), was computed as the difference between the R2 of the full model (which included PRS as an additional predictor) and that of the reference model. This approach allowed us to quantify the additional variance in LDL levels explained by the inclusion of PRS in the model. Mean LDL values across the deciles of EAS_PRS, EUR_PRS, and multi-ancestry PRS were computed in all individuals of TWB to evaluate the range of phenotypic variability cover for these PRSs.

## Results

### Study populations

In the United Kingdom Biobank, the estimated ethnic groups of the United Kingdom (United Kingdom) and China had significantly different study participant characteristics (Table 1). In comparison to people in the United Kingdom (United Kingdom), Chinese participants had lower LDL concentrations (mean, SD: 3.42 mmol/L, 0.77), lower TC levels (mean, SD: 5.54 mmol/L, 1.03), and similar HDL levels (mean, SD: 1.46 mmol/L, 0.38). They were also younger (mean age, SD: 52.3, 7.71). The Chinese participants had a lower percentage of men compared to the United Kingdom (38.8% vs. 45.9%). Participants from China had a significantly lower body mass index (BMI) (mean, SD: 24.07 kg/m2, 3.4) compared to United Kingdom participants (*p*-value < 2.2 × 10^−16^) (Supplementary Table S1).

**TABLE 1:** Study participant characteristics stratified by estimated ethnicity in the United Kingdom Biobank and Taiwan Biobank. HC, hypercholesterolemia; HDL, high-density lipoprotein cholesterol; LDL, low-density lipoprotein cholesterol; and SD, standard deviation.

	Participants, N	Males, N (%)	Age, mean (SD)	HC cases, N (%)	HC controls, N (%)	BMI, mean (SD)	LDL, mean (SD)	LDL, range (mmol/L)	HDL, mean (SD)	TC, mean (SD)
United Kingdom Biobank										
United Kingdom	423,596	194,259 (45.9)	56.81 (8.02)	110,166 (26.01)	313,430 (73.99)	27.4 (4.76)	3.57 (0.87)	0.27–9.80	1.45 (0.38)	5.71 (1.14)
Poland	4,095	1544 (37.7)	54.4 (7.53)	1088 (26.57)	3007 (73.43)	27.39 (4.96)	3.59 (0.85)	1.20–7.42	1.49 (0.4)	5.76 (1.13)
Italy	6,451	2,882 (44.7)	54.5 (8.41)	1624 (25.17)	4,827 (74.83)	27.35 (4.94)	3.56 (0.86)	0.28–7.67	1.45 (0.38)	5.68 (1.12)
Ashkenazi	2,359	1067 (45.2)	58.09 (7.1)	613 (25.99)	1746 (74.01)	27.13 (4.54)	3.55 (0.9)	1.16–8.62	1.44 (0.39)	5.68 (1.2)
Iran	1145	680 (59.4)	51.99 (7.98)	234 (20.44)	911 (79.56)	27.98 (4.55)	3.43 (0.86)	1.36–6.65	1.28 (0.33)	5.4 (1.11)
India	6,303	3413 (54.1)	53.42 (8.41)	1135 (18.01)	5168 (81.99)	27.42 (4.5)	3.35 (0.85)	0.97–6.98	1.25 (0.32)	5.31 (1.12)
Nigeria	3802	1744 (45.9)	51.95 (8.14)	551 (14.49)	3251 (85.51)	29.82 (5.31)	3.21 (0.84)	0.85–7.08	1.43 (0.35)	5.17 (1.09)
Caribbean	2,492	898 (36)	52.52 (8.13)	396 (15.89)	2096 (84.11)	29.49 (5.56)	3.28 (0.83)	1.10–6.52	1.47 (0.38)	5.29 (1.09)
China	1480	545 (36.8)	52.33 (7.71)	263 (17.77)	1217 (82.23)	24.07 (3.4)	3.42 (0.77)	1.10–7.04	1.46 (0.38)	5.54 (1.03)
Taiwan Biobank										
Taiwan	68,978	21,495 (31.2)	51.0 (10.9)	8,196 (13.5)	60,782 (86.5)	24.25 (3.8)	3.16 (0.82)	0.02–9.59	1.43 (0.35)	5.12 (0.93)

In the TWB, the percentage of men is 31.2%, which is lower than the percentage of Chinese participants in the United Kingdom Biobank, while the age distribution (mean, SD: 51.0, 10.9, respectively) is similar. In addition, TWB individuals had lower levels of lipid traits, including LDL (mean, SD: 3.16 mmol/L, 0.82), HDL (mean, SD: 1.43 mmol/L, 0.35), and TC (mean, SD: 5.12 mmol/L, 0.93), but higher BMI (mean, SD: 24.25 kg/m^2^, 3.80).

### Evaluation of the PRS in the nine estimated populations from the United Kingdom Biobank

**FIGURE 1:**
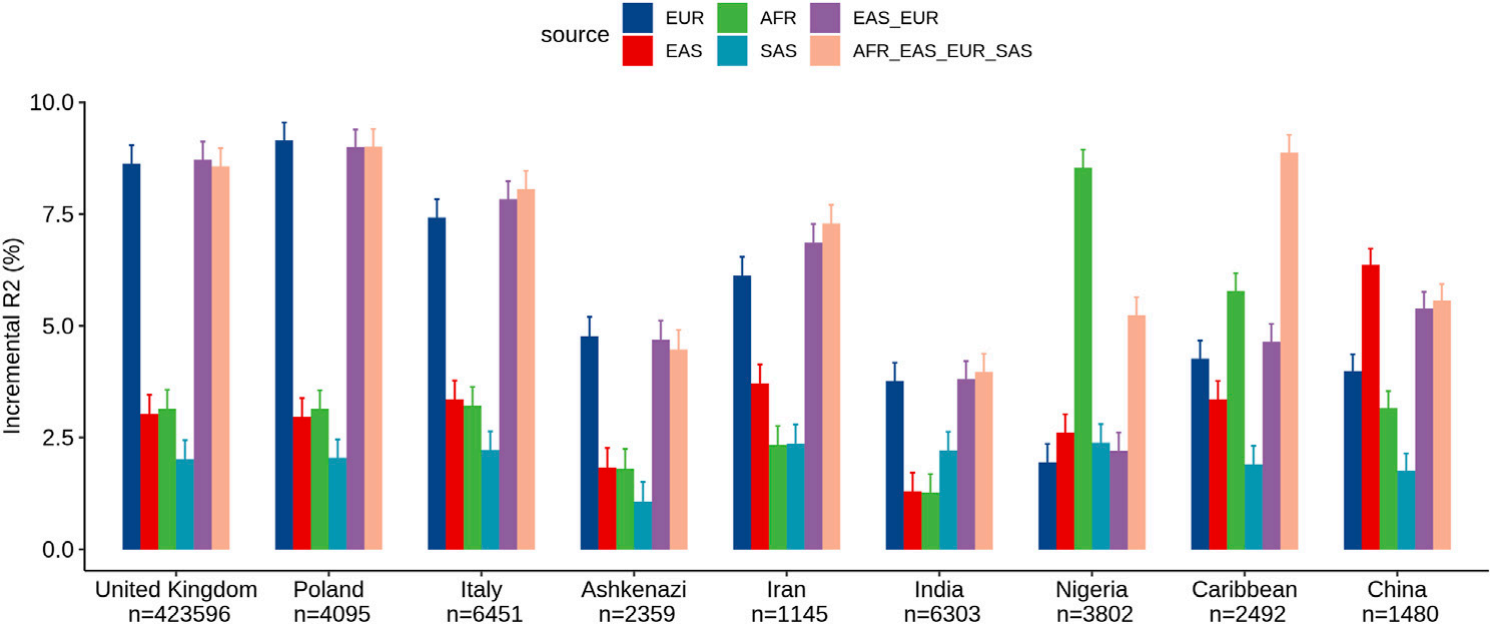
Comparison of LDL prediction performance between ancestry-specific and multi-ancestry PRS models across nine ancestry groups in the United Kingdom Biobank. Incremental R^2^ value was computed as the difference between the *R* ^2^ of the full model (which included PRS as an additional predictor along with covariates) and that of the reference model.

We assessed the performance of ancestry-specific PRS for LDL levels across the nine estimated populations in the UKB (Figure 1). As expected, the LDL PRS derived from the European GWAS (EUR_PRS) was associated with the best performance in different European populations (namely, United Kingdom, Poland, and Italy) and in Middle East populations (namely, Ashkenazi Jews and Iranians). Similarly, LDL PRS derived from the African GWAS (AFR_PRS) showed the best performance in the population of African origin (Nigeria and Caribbean). LDL PRS derived from the East Asian GWAS (EAS_PRS) was the best performing population in the Chinese population. Surprisingly, when we tested EUR_PRS and PRS derived from the South Asian GWAS (SAS_PRS) in the Indian participants, EUR_PRS performed better than SAS_PRS.

Concerning the multi-ancestry PRS, we tested a PRS derived from a meta-analysis of European and East Asian GWASs (EUR_EAS_PRS) and a global PRS derived from a meta-analysis of the four ancestries (EUR_EAS_SAS_AFR_PRS). The multi-ancestry PRS showed comparable prediction to ancestry-specific PRS and seems to be more generalizable across populations, particularly for European, Middle East, and SAS populations. For instance, for the United Kingdom population, the adjusted *R* ^2^% using EUR_PRS (8.62%) was similar to that using EUR_EAS_SAS_AFR_PRS (8.56%). For the AFR and EAS populations, ancestry-specific PRS performed better than multi-ancestry PRS. For instance, for the Chinese population, the adjusted *R* ^2^% using EAS_PRS (6.35%) was higher than that using EUR_EAS_SAS_AFR_PRS (5.55%).

### Evaluation of the PRS in the Taiwan Biobank

**FIGURE 2:**
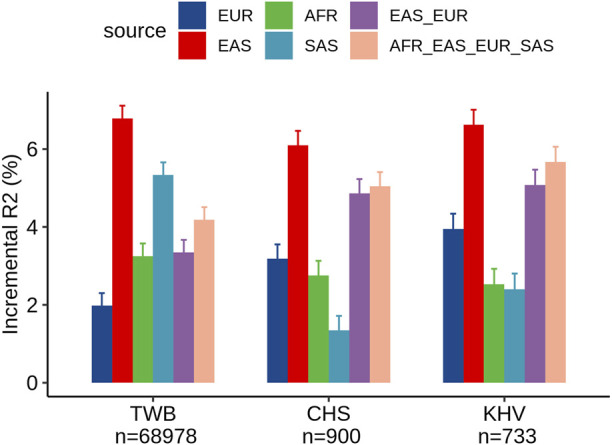
Comparison of LDL prediction performance between ancestry-specific and multi-ancestry PRS models in individuals from the Taiwan Biobank and two East Asian sub-populations of the United Kingdom Biobank (CHS and KHV).

Within the TWB, we evaluated the different ancestry-specific and multi-ancestry PRSs for LDL levels (Figure 2). Similar to our findings in UKB Chinese participants, the EAS_PRS (adjusted *R* ^2^% = 9.3%) also demonstrated better performance than EUR_PRS (adjusted *R* ^2^% = 4.5%) in the TWB individuals and had an even better performance compared to multi-ancestry PRS (adjusted *R* ^2^% = 6.7%). We also compared the performance of PRS between TWB individuals and the East Asian subpopulations from the UKB. We found that EAS_PRS has a comparable performance, particularly between populations from the TWB (adjusted *R* ^2^% = 6.5%) and CHS (adjusted *R* ^2^ = 6.1%) from the UKB. We conducted an analysis and calculated the raw R2 only for the PRS in the nine groups, and the results appear to align with the incremental R^2^ value (Supplementary Figures S1, S2).

### Association between different PRS strata and LDL values

**FIGURE 3:**
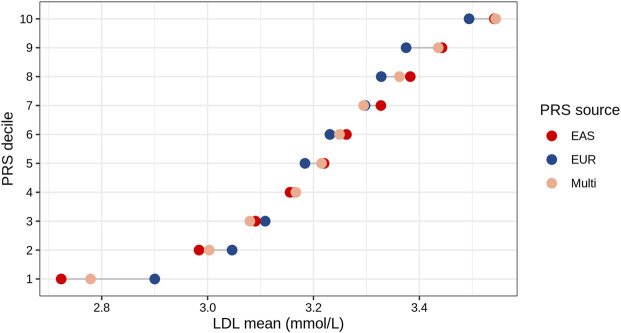
LDL mean values across the deciles of EAS, EUR, and multi-ancestry PRSs of East Asians (Taiwan Biobank).

We analyzed the mean of LDL levels in individuals from the TWB based on their EAS_PRS, EUR_PRS, and multi-ancestry PRS deciles. We compared the difference in mean LDL levels between the lowest and highest deciles of EAS_PRS, EUR_PRS, and multi-ancestry PRS. Our findings showed that in East Asians, EAS_PRS explained a wider range of phenotypic variability compared to EUR_PRS. Particularly, the difference in mean LDL levels between the lowest and highest EAS_PRS deciles was 0.82, while that for EUR_PRS, it was 0.59 (Figure 3). The mean difference in LDL levels between the lowest and highest multi-ancestry PRS deciles was 0.76. However, the mean LDL levels in the highest deciles in both EAS_PRS and multi-ancestry PRS were the same (LDL mean (mmol/L) = 3.54, *p* = 0.86).

## Discussion

This study aimed at predicting LDL in two EAS populations (from UKB and TWB) using the latest GWAS. Our findings indicate that 1) ancestry-specific PRS yield better performance in predicting LDL levels, and 2) multi-ancestry PRSs together with computational approaches integrating a population-specific LD pattern can be used to enhance the generalizability of PRSs. In particular, the multi-ancestry PRSs showed that even the relatively small proportions of non-European samples can significantly improve predictions in non-EUR populations. Our work emphasizes the importance of conducting GWAS that include diverse populations to enhance the generalizability of PRSs, even when the availability of diverse population samples is limited.

The findings presented indicate distinctions in the predictive power of PRS based on different ancestry groups when examining LDL cholesterol levels across diverse populations. As expected, the European-specific PRS (EUR_PRS) showed superior performance in all European populations. Similarly, we observed the same pattern in Middle Eastern populations and South Asians, further extending the applicability of the EUR_PRS. Interestingly, the EUR_PRS outperformed the South Asian PRS (SAS_PRS) in the Indian participants, though this could be also influenced by the difference sample size of the population-specific GWAS. We observed varying LDL prediction accuracies between United Kingdom and Taiwan Biobanks using SAS-based GWAS, and this is influenced by lifestyle, sample size, or gene–environment interactions. Ancestry-specific PRSs often outperformed target population PRS (e.g., EAS_PRS for the Chinese population and AFR_PRS for African origins), highlighting the importance of tailored genetic studies. These findings corroborate the need of multi-ancestry genetic data in enhancing the accuracy and precision of risk predictions.

Our targeted analysis in East Asian demonstrated that the difference in mean LDL levels between the lowest and highest deciles for the EAS_PRS was notably higher than the differences observed for the EUR_PRS and the multi-ancestry PRS. This suggests that the EAS_PRS might have a stronger discriminatory power for LDL cholesterol levels among East Asians compared to EUR_PRS. Furthermore, the similar mean LDL values observed in the top decile for multi-ancestry PRS and EAS_PRS (with a *p*-value of 0.86 indicating no significant difference between them) is of particular interest. This similarity suggests that multi-ancestry PRSs including relatively small proportions of non-European samples may improve the prediction of high LDL levels in East Asians.

Our study further suggest that statistical genetics approaches can be used to take advantage of the already available global GWAS data, even when the number of non-European samples is limited. One example, the latest GWAS includes individuals across five genetic ancestry groups: admixed African or African (6.0% of the sample), East Asian (8.9%), European (79.8%), Hispanic (2.9%), and South Asian (2.5%) (Graham et al., 2021). Recently published Bayesian PRS approaches demonstrated an improvement in the accuracy of PRSs in non-European populations by utilizing common genetic effects across ancestries (Ruan et al., 2022; Hoggart et al., 2023). Another recent study, the authors conducted a benchmarking analysis to compare several PRS methods for multi-ancestry analysis in the UKB dataset, which included lipid traits and EAS data using GWAS data as well (Zhang et al., 2023). The findings of this study provided insights on the use of statistical methods to improve prediction performance in non-Europeans.

The applicability of the findings on the portability of PRS from multi-ancestry meta-analyses to other traits needs to be taken into account, considering multiple factors (Majara et al., 2023). These factors include the heritability of the trait (Momin et al., 2023), genetic correlation (Shi et al., 2021), causal variants allele frequencies (Cavazos and Witte, 2020), gene-environment interactions (Peterson et al., 2019), and the inclusion of multi-ancestry populations in GWAS (Fatumo et al., 2022; Yengo et al., 2022). In a recent study, they estimated the cross-ancestry genetic correlation for cholesterol and observed a significant genetic heterogeneity between ancestries for total and LDL cholesterol (Momin et al., 2023). While many traits exhibit a significant shared genetic correlation across ancestries, indicating the potential transferability of multi-ancestry PRS (Ho et al., 2020), some traits have specific genetic variations that are more commonly found in particular ancestral groups (El-Boraie et al., 2021; Kamiza et al., 2022). To ensure the effective use of PRS in diverse populations, it is crucial to conduct comprehensive investigations considering these factors and include a representative range of ancestries in future GWAS studies (Duncan et al., 2019). Moreover, a recent study emphasizes the necessity of moving away from discrete genetic ancestry clusters and embracing the continuum of genetic ancestries when analyzing and interpreting PRS (Ding et al., 2023). By accounting for individual variation and considering the diverse genetic backgrounds within populations, more accurate PRS assessments can be achieved.

By leveraging the available diverse GWAS data, we can improve the generalizability of PRSs and ultimately enhance our ability to predict complex disease risk across diverse populations. As such, our study provides valuable insights into the development and implementation of PRSs for predicting lipid traits in East Asian populations and highlights the need for continued efforts to increase diversity in genetic research while also working on bioinformatics approaches to meta-analyze the association signal across different populations.

## Conclusion

In our study, we evaluated the performance of ancestry-specific and multi-ancestry PRSs for LDL in various populations, including East Asians from the United Kingdom Biobank and Taiwan Biobank. The findings corroborated that ancestry-specific PRSs performed better than the target population PRSs in their respective ancestries. In particular, EAS_PRS had better performance in East Asian populations, while EUR_PRS showed better performance in European and Middle East populations. The multi-ancestry PRS analysis showed that even a small proportion of non-European samples can significantly improve the prediction in non-EUR populations. These findings provide valuable insights into the development of PRSs for diverse populations and the potential clinical applications of PRSs. On one hand, our analysis suggests that incorporating cross-ancestry GWAS data and utilizing optimized computational algorithms to account for population-specific LD-patterns can improve the generalizability of PRS. On the other hand, these results further emphasize the necessity of enhancing genetic diversity in GWASs and establishing large-scale population-based cohorts to more accurately model the genetic liability of multifactorial traits, such as LDL cholesterol.

## Data Availability

Publicly available datasets were analyzed in this study. These data can be found at: http://www.ukbiobank.ac.uk/about-biobankuk/ and https://www.biobank.org.tw/. The codes related to the statistical analysis for this study have been deposited on GitLab, and the generated ancestry-specific and multi-ancestry PRS weights for LDL (excluding UK Biobank samples) are available on Zenodo at the following location (doi:10.17881/8wqn-x712).

## Abbreviations

AFR, African; CHS, Han Chinese South; EUR, European; EAS, East Asian; GWAS, genome-wide association study; KHV, Kinh in Ho Chi Minh City, Vietnam; LDL-C, low-density lipoprotein cholesterol; PCA, principal component analysis; PRS, polygenic risk score; SAS, South Asian; TWB, Taiwan Biobank; TC, total cholesterol; UKB, United Kingdom Biobank.
