# Supplementary material for: Trans-ancestry polygenic models for the prediction of LDL blood levels: an analysis of the United Kingdom Biobank and Taiwan Biobank
Source: Front Genet. 2023 Nov 23;14:1286561. doi: 10.3389/fgene.2023.1286561 (PMC10704094; doi:10.3389/fgene.2023.1286561)
Supplement: Supplementary file 1 [file DataSheet1.docx]

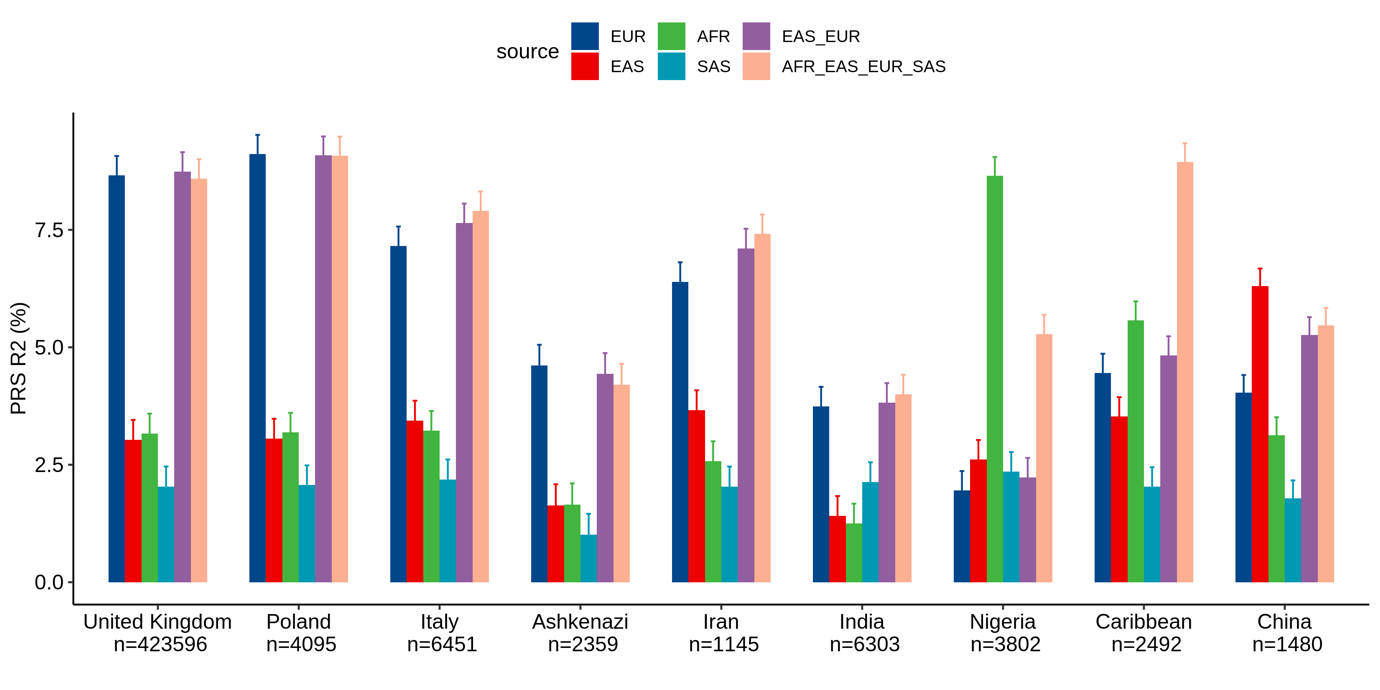


Figure S1. Comparing LDL Prediction Performance between Ancestry-Specific and Multi-Ancestry PRS Models across nine ancestry groups in the UK Biobank. R2 was computed of the model that only includes the PRS.


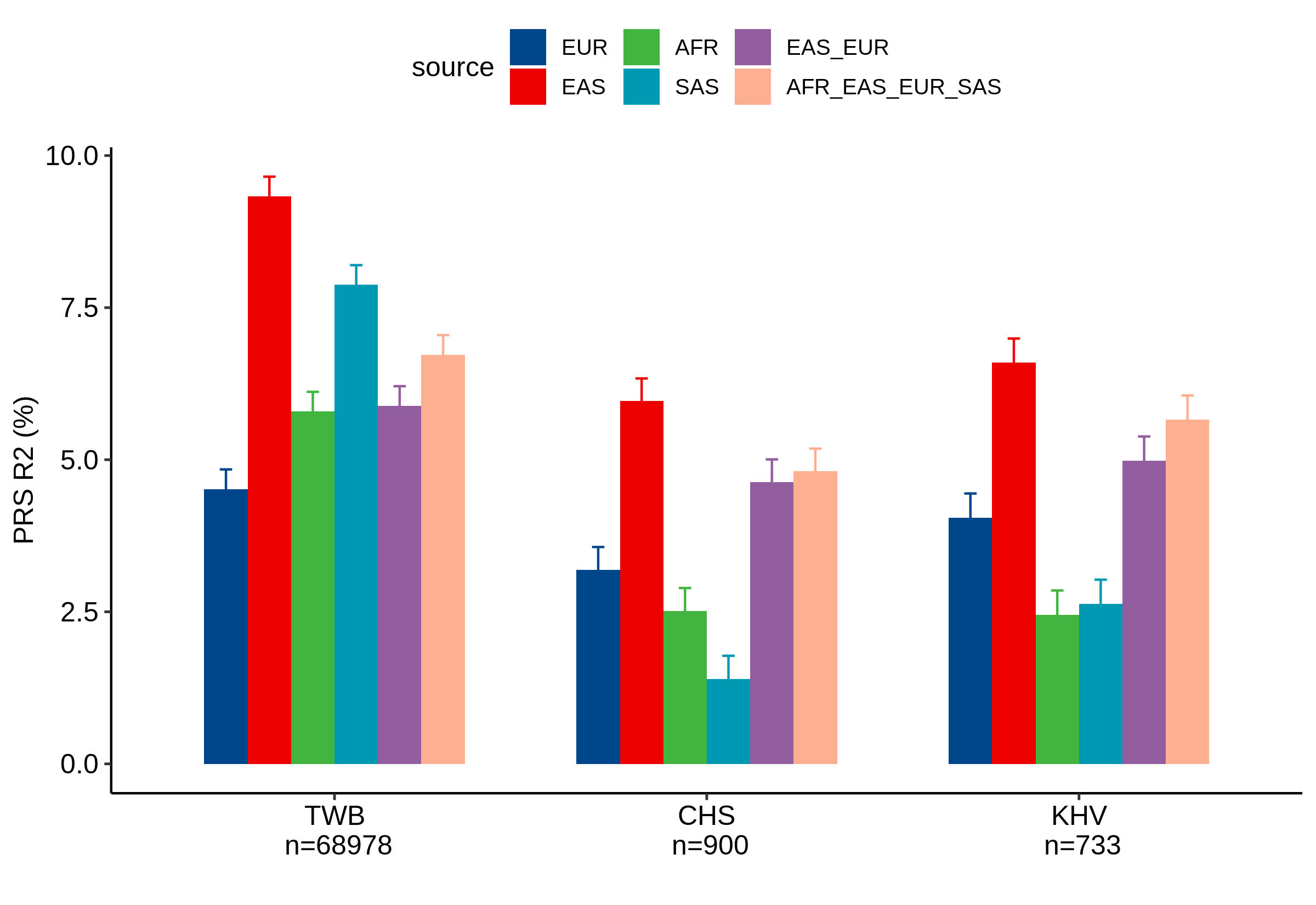


Figure S2. Comparing LDL Prediction Performance between Ancestry-Specific and Multi-Ancestry PRS Models in Individuals from the Taiwan Biobank and Two East Asian Sub-Populations of the UK Biobank (CHS, KHV). R2 was computed of the model that only includes the PRS.

|  | Sex | Age | BMI | LDL | HDL | TC |
| --- | --- | --- | --- | --- | --- | --- |
| United Kingdom/China | <2.2 x 10^-16^ | <2.2 x 10^-16^ | <2.2 x 10^-16^ | <1.8 x 10^-13^ | 0.53 | <1.6 x 10^-10^ |
| Poland/China | <2.2 x 10^-16^ | <2.2 x 10^-16^ | <2.2 x 10^-16^ | <8.5 x 10^-13^ | 0.03 | <1.0 x 10^-11^ |
| Italy/China | <2.2 x 10^-16^ | <2.2 x 10^-16^ | <2.2 x 10^-16^ | <2.4 x 10^-10^ | 0.26 | <2.5 x 10^-6^ |
| Ashkenazi/China | <2.2 x 10^-16^ | <2.2 x 10^-16^ | <2.2 x 10^-16^ | <1.6 x 10^-06^ | 0.14 | <1.9 x 10^-5^ |
| Iran/China | 0.0001 | 0.26 | <2.2 x 10^-16^ | 0.68 | <2.2 x 10^-16^ | 0.001 |
| India/China | <2.2 x 10^-16^ | <4.5 x 10^-6^ | <2.2 x 10^-16^ | 0.003 | <2.2 x 10^-16^ | <7.3 x 10^-14^ |
| Nigeria/China | <2.2 x 10^-16^ | 0.11 | <2.2 x 10^-16^ | <2.2 x 10^-16^ | 0.03 | <2.2 x 10^-16^ |
| Caribbean/China | <2.2 x 10^-16^ | 0.46 | <2.2 x 10^-16^ | <1.48 x 10^-7^ | 0.39 | <1.5 x 10^-12^ |

Table S1: Comparison of participant characteristics across all ancestry groups against China participants in the UK Biobank. For continuous variables, p-values from the Welch t-statistic tests are reported, while for categorical and binary variables, p-values from Pearson’s Chi-squared tests are reported. HC, hypercholesterolemia; HDL, high-density lipoprotein cholesterol; LDL, low-density lipoprotein cholesterol; SD, standard deviation.
